# The α isoform of topoisomerase II is required for hypercompaction of mitotic chromosomes in human cells

**DOI:** 10.1093/nar/gku076

**Published:** 2014-01-28

**Authors:** Christine J. Farr, Melissa Antoniou-Kourounioti, Michael L. Mimmack, Arsen Volkov, Andrew C. G. Porter

**Affiliations:** ^1^Department of Genetics, University of Cambridge, Downing St, Cambridge CB2 3EH, UK and ^2^Centre for Haematology, Faculty of Medicine, Imperial College London, Hammersmith Hospital Campus, Du Cane Rd, London W12 0NN, UK

## Abstract

As proliferating cells transit from interphase into M-phase, chromatin undergoes extensive reorganization, and topoisomerase (topo) IIα, the major isoform of this enzyme present in cycling vertebrate cells, plays a key role in this process. In this study, a human cell line conditional null mutant for topo IIα and a derivative expressing an auxin-inducible degron (AID)-tagged version of the protein have been used to distinguish real mitotic chromosome functions of topo IIα from its more general role in DNA metabolism and to investigate whether topo IIβ makes any contribution to mitotic chromosome formation. We show that topo IIβ does contribute, with endogenous levels being sufficient for the initial stages of axial shortening. However, a significant effect of topo IIα depletion, seen with or without the co-depletion of topo IIβ, is the failure of chromosomes to hypercompact when delayed in M-phase. This requires much higher levels of topo II protein and is impaired by drugs or mutations that affect enzyme activity. A prolonged delay at the G2/M border results in hyperefficient axial shortening, a process that is topo IIα-dependent. Rapid depletion of topo IIα has allowed us to show that its function during late G2 and M-phase is truly required for shaping mitotic chromosomes.

## INTRODUCTION

Vertebrates have two topoisomerase (topo) II isoforms: α and β, that are encoded by separate genes (1–3). The two forms have distinct patterns of expression: topo IIα is cell cycle-regulated and is essential for the survival of proliferating cells (4–7). It accumulates on chromatin during M-phase (8), a dynamic localization (9,10) that is dependent on its C-terminal domain (11). In contrast, topo IIβ is expressed throughout the cell cycle and in postmitotic cells but is dispensable at the cellular level (3,9,12–17) and localizes to mitotic chromatin only weakly (9–11). Topo IIβ is not normally able to compensate for loss of IIα, although it has been shown that cultured human cells can be rescued from the lethal effects of IIα depletion by IIβ if levels of the β isoform are high (11).

Although topo IIα is the major form of topo II responsible for decatenation, mitotic chromosome formation and chromosome segregation in proliferating cells, the contribution of the two isoforms has not yet been fully established (18,19). While data from some model systems have shown topo II to be essential in mitotic chromosome compaction, other studies have been equivocal (20–24). Genetic analyses suggest that topo II is required for chromosome condensation in *Schizosaccharomyces pombe* (25) but not in *Sacchromyces cerevisiae* (26). *In vitro* studies of chromosome condensation in mitotic extracts (27–31) in which topoII is immunodepleted or inactivated by inhibitors showed varying requirements for topo II, from absolute dependence (29) to redundant (28). Many *in vivo* studies in higher eukaryotes have made use of topo II inhibitors, such as the bisdioxopiperazines (e.g. ICRF-193) (32–38). Such studies generally support a role for topo II in chromosome condensation, but again condensation was impaired to varying degrees. Moreover, the interpretation of these experiments is complicated by the dominant toxic effects that arise from ICRF-193 not only blocking the catalytic cycle but also trapping the topo II dimer on DNA as a closed protein clamp (39) that perturbs chromatin structure (40).

Approaches depleting both topo II isoforms, using small interfering RNA (siRNA), have suggested that this leads to poor chromosome condensation (41,42) with longer thinner chromosomes than normal. In a conditional null mutant, HT1080 cell line generated by gene targeting (7) (in which topo IIα transcription is regulatable using doxycycline) mitotic chromosome condensation occurs following topo IIα depletion by >99%, but with slower than normal kinetics, producing higher than normal levels of partially condensed chromosomes. Conditional depletion through short hairpin RNA (shRNA) targeted against chicken topo IIα in DT40 cells also produces cells with chromosomes that are longer, and thinner, than normal (43,44). Moreover, the longer thinner topo IIα-depleted mitotic chromosomes retain both SMC2 (condensin) and their intrinsic structure (based on an *in vitro* assay) (44,45). Thus, although there is clear evidence that topo II is involved in the formation of mitotic chromosomes, the phenotype seen when topo IIα, the major isoform associated with mitotic chromatin, is depleted is surprisingly mild. Therefore, we have reexamined the contribution of both topo IIα and IIβ, individually and together, to mitotic chromosome formation.

## MATERIALS AND METHODS

### Antibodies

Primary antibodies used for immunoblotting were anti-human topoisomerase IIα (mbl) (1:5000), anti-human topoisomerase IIβ (BD) (1:2000), anti-GFP (Roche) (1:2000), anti-HSP70 (Santa Cruz) (1:4000), anti-myc (abcam) (1:2000) and anti-α-tubulin (abcam) (1:10 000). Secondary antibodies were IRDye 800CW goat anti-mouse IgG (H+L) (LI-COR) (1:7000) and poly-HRP goat anti-mouse (Thermo Scientific) (1:15 000). For indirect immunofluorescence, antibodies used were anti-human topoisomerase IIα (mbl) and anti-human topoisomerase IIβ (BD) (both at 1:500). Secondary antibody used was rabbit anti-mouse FITC (Dako) (1:200).

### Cell lines

HTETOP is an HT1080-derived conditional null mutant for topoisomerase IIα (7). Transcription of the transgene encoding untagged human topo IIα is repressed using doxycycline. T2A:YFP-1, T2B:YFP-1, T2B:YFP-2 and T2B:YFP-3 are HTETOP clones rescued from dox-induced lethality by expression of yellow fluorescent protein (YFP)-tagged topoisomerase IIα and IIβ (11). All other cell lines described have been generated from HTETOP during the course of this work.

### Cell culture, transfections and drug treatments

The HT1080-derived cell lines were grown routinely in Dulbecco’s modified Eagle’s medium containing glutamax, 10% foetal bovine serum, penicillin and streptomycin (all from Invitrogen-Gibco) at 37°C. To repress the Tet-regulatable topo IIα transgene, cells were grown in medium containing 1 µg/ml doxycycline (dox) (Sigma), with the medium renewed every third day. To trigger degradation of AID-tagged proteins, either, a synthetic auxin, 1-naphthaleneacetic acid (NAA) (Sigma-Aldrich) [dissolved in dimethyl sulfoxide (DMSO) immediately before use] or, a natural auxin, indole-3-acetic acid (IAA) (Sigma-Aldrich) (dissolved in H_2_O immediately before use) was added to the medium (final concentrations: 0.5–1 mM). For stable transfection of DNA, cells were electroporated using standard conditions: 6 × 10^6^ cells in 800 µl of Dulbecco’s phosphate-buffered saline [DPBS] using a BioRad Pulser II with 30 µg of linearized plasmid DNA at 250 µF, 400 V and 200 Ω. After 24–48 h, selection was applied. Biochemical selections were as follows: puromycin (Sigma), 0.5 µg/ml; blasticidin S (MP Biomedicals), 3 µg/ml. The siRNA (100 pm) (MWG) was transiently transfected into ∼250 000 cells using RNAiMAX (Invitrogen) in 6-well dishes according to the manufacturer’s protocol. The siRNA used against topo IIβ in HT1080 cells was as previously described: GGAUUUAUGUGGUAGAUCAA (42). To chemically inhibit topoisomerase II, ICRF-193 (EuroMedex) (dissolved in DMSO) was added at a final concentration of 2 µg/ml. Nocodazole (Calbiochem) was added at a final concentration of 50 ng/ml and RO3306 (Calbiochem) at 5 µM.

### Expression constructs

To generate expression constructs encoding Flag-tagged wild-type (WT) and mutant (K662R) topo IIα, the pcDNA3 vector (Invitrogen) was modified to carry a puromycin-resistance cassette (from pPUR Clontech) in place of the SV_2_neo^r^ cassette (designated pcDNA3-puro). Three copies of the Flag epitope were cloned, into the multicloning site, using HindIII and EcoRI. Lastly, the open reading frame (ORF) of human topo IIα was introduced as an EcoRV-NotI fragment. The QuikChange site-directed mutagenesis kit was used to generate specific point mutations according to the manufacturer’s instructions (Stratagene).

For ectopic expression of the rice TIR1 F-box protein, pOsTIR1:9myc was generated by digestion of pAID1.1N (also known as pNHK60) (46) with EcoRV and Sma1 (to remove the IRES and degron) followed by religation. For expression of human topo IIα, tagged at its N-terminus with the AID degron followed by three copies of the Flag epitope, expression plasmid pCPFAT was generated through the following series of manipulations: (i) oligonucleotides encompassing three copies of the Flag epitope were cloned into the polylinker of Bluescript (pBS SK) using EcoRI and HindIII; (ii) the degron was PCR amplified from pAID1.2N and cloned into the modified Bluescript plasmid using Asp718 and HindIII; (iii) both the degron and Flag epitope were then transferred as an Asp718-EcoRI fragment into pcDNA3-puro; and (iv) lastly, the ORF for human topo IIα was transferred as an EcoRV-NotI fragment resulting in an ORF encoding AID:Flag:hTopo IIα as a fusion protein.

### Assessment of mitotic chromosome formation

Cells were plated onto SuperFrost (VWR) slides in chambers 24 h before processing. Hypotonic treatment (75 mM KCl, 10 min at 37°C) was applied before fixation in ice-cold 3:1 methanol: acetic acid (Carnoy’s Fix). The fixation step was repeated three more times and the slides air-dried. DNA was counterstained with 4′6-Diamidino-2-Phenylindole (DAPI) (0.5 µg/ml) and the slides mounted in Vectorshield (Vector Labs). Images were captured using the AF6000 system with a Leica DM6000B upright fluorescence microscope.

### Indirect immunofluorescence and microscopy

Cells were grown overnight on SuperFrost (VWR) slides and fixed in PTEMF for 10 min (20 mM Pipes pH 6.8, 0.2% Triton X-100, 10 mM EGTA, 1 mM MgCl_2_, 4% paraformaldehyde). Blocking (10% foetal bovine serum), antibody dilutions and washes were all undertaken in DPBS Tween 20 (0.05%). DNA was counterstained with DAPI (0.5 µg ml^−^^1^) and mounted in Vectorshield (Vector Labs). Images were captured using the AF6000 system with a Leica DM6000B upright fluorescence microscope.

### Immunoblotting

Cells were harvested by trypsinization, washed and snap frozen. Pellets were lysed in CelLytic M (Sigma) containing protease-inhibitor cocktail (Roche Complete Mini) according to the manufacturer’s recommendations. Lysates were cleared by centrifugation at 13 000*g* and fractionated by sodium dodecyl sulfate–polyacrylamide gel electrophoresis (Xcell SureLock Mini-Cell system, Invitrogen) using Tris-glycine running buffer. Gels were transferred in buffer containing 20% methanol, without sodium dodecyl sulfate. For fluorescence immunoblotting, PVDF-FL membrane was used with the blocking and primary antibody steps performed in Odyssey blocking buffer (LI-COR Biosciences): Tris-buffered saline with 0.1% Tween-20 (TBST) (1:1). Secondary antibody incubation was in TBST plus 5% powdered milk. Fluorescence intensities were determined using a charge coupled device (CCD) scanner (Odyssey; LI-COR Biosciences) according to the manufacturer’s instructions. For chemiluminescent detection, PVDF-P membrane was used, with all steps being performed in TBST plus milk (2–5%). Antibody–antigen complexes were detected using ECL Plus according to the manufacturer’s instructions (GE Healthcare).

### Statistical analyses

A two-tailed *t*-test (unpaired) was used to assess the statistical significance of the observed differences in levels of axial shortening data in Figures 2C, 3 and 4C. The *P*-values expressed as *P ≤ 0.05, **P ≤ 0.005 and ***P ≤ 0.0005 were considered significant.

## RESULTS

### The impact of depletion of both topo 2 isoforms on mitotic chromosome formation

**Figure 1.**
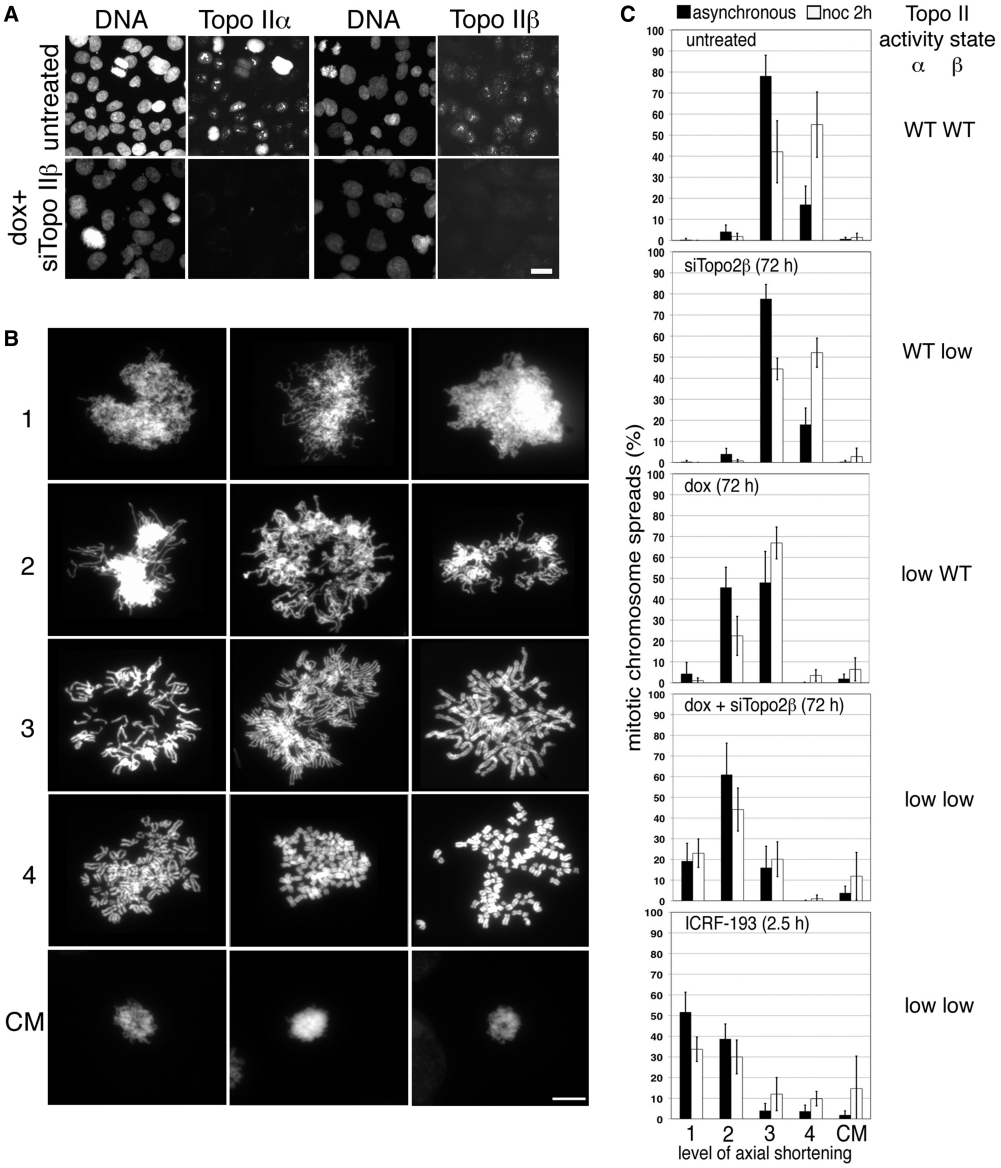
The effect of depleting the two topo II isoforms on mitotic chromosome formation. (**A**) Indirect IF of topo IIα and topo IIβ in HTETOP cells either untreated or exposed to dox (topo IIα depletion) + siTopo IIβ for 3 days. Cells were fixed *in situ* using PTEMF. Topo II was detected using either anti-topo IIα or anti-topo IIβ antibody (FITC) and DNA counterstained using DAPI. Scale bar, 25 µm. (**B**) Representative images of DAPI-stained chromosome spreads assigned to various levels (1–4) of axial shortening are shown, together with examples of compact chromatin masses (CM). Scale bar, 10 µm. (**C**) Frequencies of the various levels of axial shortening observed in mitotic HTETOP cells expressing normal levels of both topo II isoforms compared with cells depleted of either topo IIα or IIβ, or both, over 72 h, or cells in which both isoforms have been chemically inhibited for 2.5 h (ICRF-193). Cells were grown on slides overnight, treated with hypotonic (75 mM KCl, 10 min) before fixation in ice-cold methanol: acetic acid and examined after DAPI staining of the DNA. Data were collected both from asynchronously growing populations and from cells arrested in M-phase (nocodazole 2 h). Data points represent the mean (±standard deviation (sd)) based on ≥3 independent experiments, with ∼100 cells scored per experiment.

The HTETOP cell line was derived from HT1080 through the targeted disruption of both endogenous topo IIα alleles (7). The cells are kept alive through expression of topo IIα from a doxycycline (dox)-regulatable transgene: in the presence of dox, transcription is repressed and after 72 h, the amount of topo IIα protein falls to ∼1% of the starting level, with all cells eventually dying (7,45). Where simultaneous depletion of both isoforms is required, transient siRNA can be used to target topo IIβ and indirect immunofluorescence (IF) and chemiluminescent immunoblotting used to confirm effective depletion (Figure 1A and data not shown).

Chromosome condensation was examined in chromosome spreads from HTETOP cells treated with doxycycline (72 h), siTopo IIβ (72 h), ICRF-193 (2.5 h) and nocodazole (2 h) in various combinations. Cells were grown on slides overnight, treated with 75 mM KCl for 10 min, fixed with cold methanol/acetic acid and examined after DAPI staining of the DNA. The extent of mitotic chromosome formation for each spread was scored from 1 (lowest) to 4 (highest) as follows: level 1—entangled chromatin with little evidence of longitudinal shortening of chromosomes; level 2—evidence of longitudinal shortening, with some chromosome arms evident, but still entangled and lacking sister chromatid resolution; level 3—individualized chromosomes with some sister chromatid resolution evident (typical of asynchronously-growing topo IIα-expressing cells); and level 4—hypercompaction, with short wide chromosomes and good sister chromatid resolution (typical of normal cells delayed in M-phase by spindle poisons) (Figure 1B). As we have reported previously (7), cells depleted of topo IIα have a significantly increased frequency of longer thinner mitotic chromosomes, with >40% of cells showing only level 2 compaction. Moreover, few of these cells achieve hypercompaction (level 4) when delayed in M-phase using nocodazole (Figure 1C): <5%, compared with >50% of cells expressing normal levels of topo IIα. Depletion of topo IIβ alone does not have any noticeable impact, but its depletion in cells also depleted of topo IIα results in a stronger phenotype than that seen for topo IIα depletion alone; in doubly depleted cells, 80% have chromosomes that display only level 1 or 2 compaction. Chemical inhibition of both isoforms using ICRF-193 produces the most severe perturbation of mitotic chromosome formation (Figure 1B). Occasionally cells were observed in which some compaction appeared to have occurred but in the absence of chromosome individualization, giving rise to a compact chromatin mass (CM) (Figure 1B). Such cells were generally rare (∼1%), but their frequency increased when cells from which both topo II isoforms had been depleted, or chemically inhibited, were arrested in M-phase. Under these conditions frequencies ranging from 5 to 15% were observed.

### Topo IIβ and mitotic chromosome formation

**Figure 2.**
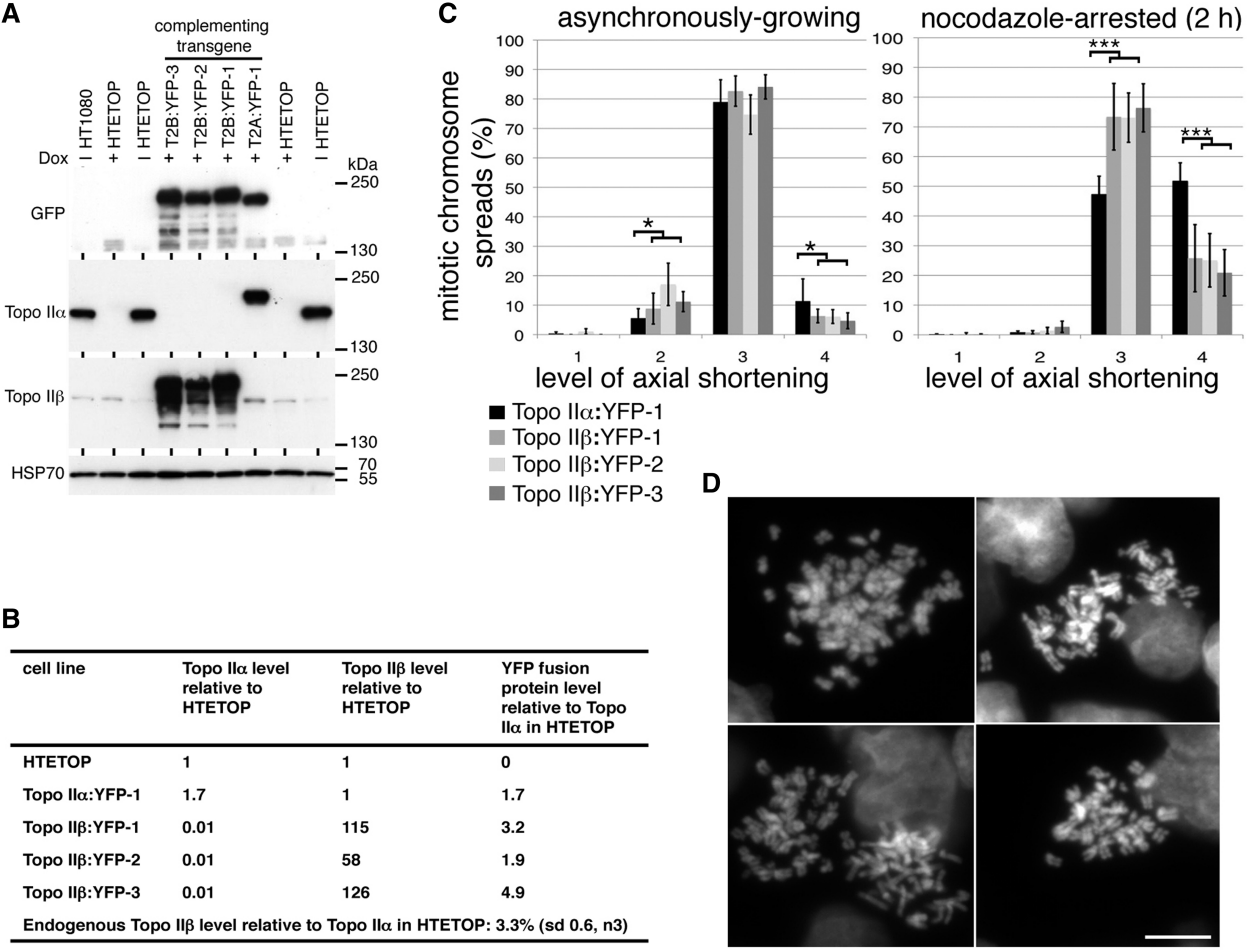
Topo IIβ and mitotic chromosome formation. (**A**) Chemiluminescent immunoblot of HTETOP clones rescued from dox lethality by expression of YFP-fused topo IIα or IIβ. Whole cell lysates of the untransfected HTETOP parental cell line (grown in the absence or presence of dox), and dox-resistant HTETOP transfectant clones expressing topo II:YFP fusion proteins (+ dox) were subjected to sodium dodecyl sulfate–polyacrylamide gel electrophoresis (5% gels) and immunoblotting. Blots were hybridized with antibodies against GFP/YFP, human topo IIα, human topo IIβ and HSP70 (loading control). (**B**) Topo IIα and IIβ levels estimated by fluorescence immunoblotting of the various cell lines. Antibodies against topo IIα and IIβ were used to estimate levels of the two isoforms in the various transfectants. YFP-immunoblotting was then used to compare levels of the fusion protein in transfectants and determine topo IIα and IIβ levels relative to the parental. (**C**) Assessment of mitotic chromosome formation in three independent HTETOP transfectant clones expressing topo IIβ:YFP compared with a reference clone expressing topo IIα:YFP. Data were collected both from asynchronously growing and nocodazole-arrested (2 h) cell populations. Data points for each cell line represent the mean (±sd) based on ≥3 independent experiments, with ∼100 cells scored per experiment. The frequency of spreads showing the compact mass (CM) phenotype were <1% in all cases. (**D**) Representative DAPI-stained chromosome spreads showing level 4 axial shortening from cells in which ∼99% of topo II is topo IIβ (YFP-tagged and the endogenous untagged isoform). Scale bar, 10 µm.

Three stable HTETOP-derived clones, rescued from dox lethality by expression of topo IIβ:YFP, were examined alongside a line rescued by expression of topo IIα:YFP (11). The levels of topo IIβ:YFP fusion protein in these clones have been shown previously to be much higher than the endogenous topo IIβ level (11). Using a combination of chemiluminescence and quantitative fluorescence immunoblotting, we examined levels of the two isoforms in the parental HTETOP cell line compared with the various lines expressing a complementing topo II:YFP transgene (Figure 2A, B). From this we estimate that the endogenous topo IIβ level in parental HTETOP cells (minus dox) is ∼3% that of topo IIα, while the amount of topo IIβ:YFP fusion protein in the rescued lines is 58–126× the endogenous topo IIβ level. Mitotic chromosome formation was examined in chromosome spreads from the various topo II:YFP expressing clones (grown continuously in dox). This revealed that asynchronously growing cells expressing topo IIβ:YFP as their (virtually) only form of topo II show a similar distribution across the various levels of chromosome compaction as topo IIα:YFP-expressing cells, although with slightly more achieving only level 2 compaction and slightly fewer displaying hypercompaction. When cells are held in M-phase with nocodazole (2 h), some topo IIβ-only expressing cells display hypercompacted chromosomes (20–25%) (Figure 2C, D). However, the frequency was always significantly lower than for cells rescued by topo IIα:YFP (∼50%) (Figure 2C).

### The effect on mitotic chromosome formation of mutating the catalytic domain residue K662

Lysine 662 of human topo IIα is a highly conserved residue within the catalytic domain that is directly involved in binding G-segment DNA (47). In *S.cerevisiae,* top2 in which the equivalent residue, K651, was substituted by alanine was unable to complement temperature-sensitive top2 mutant strains (48). From *in vitro* studies, it was concluded that whilst this mutation results in reduced relaxation activity, Lys-651 does not play a major role in the catalysis of DNA breakage and rejoining (48). In *Xenopus laevis,* mutation of the equivalent lysine to arginine (K660R) has been shown to compromise topo IIα activity in kinetoplast decatenation assays (49).

Transfections revealed that human topo IIα-K662R can rescue HTETOP cells from dox lethality. Topo IIα levels in various clones were assessed using fluorescence immunoblotting (data not shown), allowing the identification of stable transfectants with levels similar to those in the untreated parental HTETOP cell line. Consistent with the reports that mutation of this lysine interferes with the enzyme’s catalytic cycle, human clones expressing topo IIα-K662R in place of WT have a longer population doubling (PD) time than the parental line [three independent clones were found to have a PD time of 30.3 ± 4.7 h (n10), compared with 21.5 ± 1.2 h (n3) for HTETOP (mean, sd)]. Moreover, topo IIα-K662R stable cell lines display significantly higher levels of lagging chromosomes and chromatin bridges in anaphase (missegregation rate of three stable cell lines expressing Flag-tagged topo IIα-K662R was 44% ± 6.8 (mean, sd, n21), compared with 21.4% ± 6.5 (mean, sd, n21) in three stable lines expressing Flag-tagged topo IIα-WT (*P =* 0.0001). 

**Figure 3.**
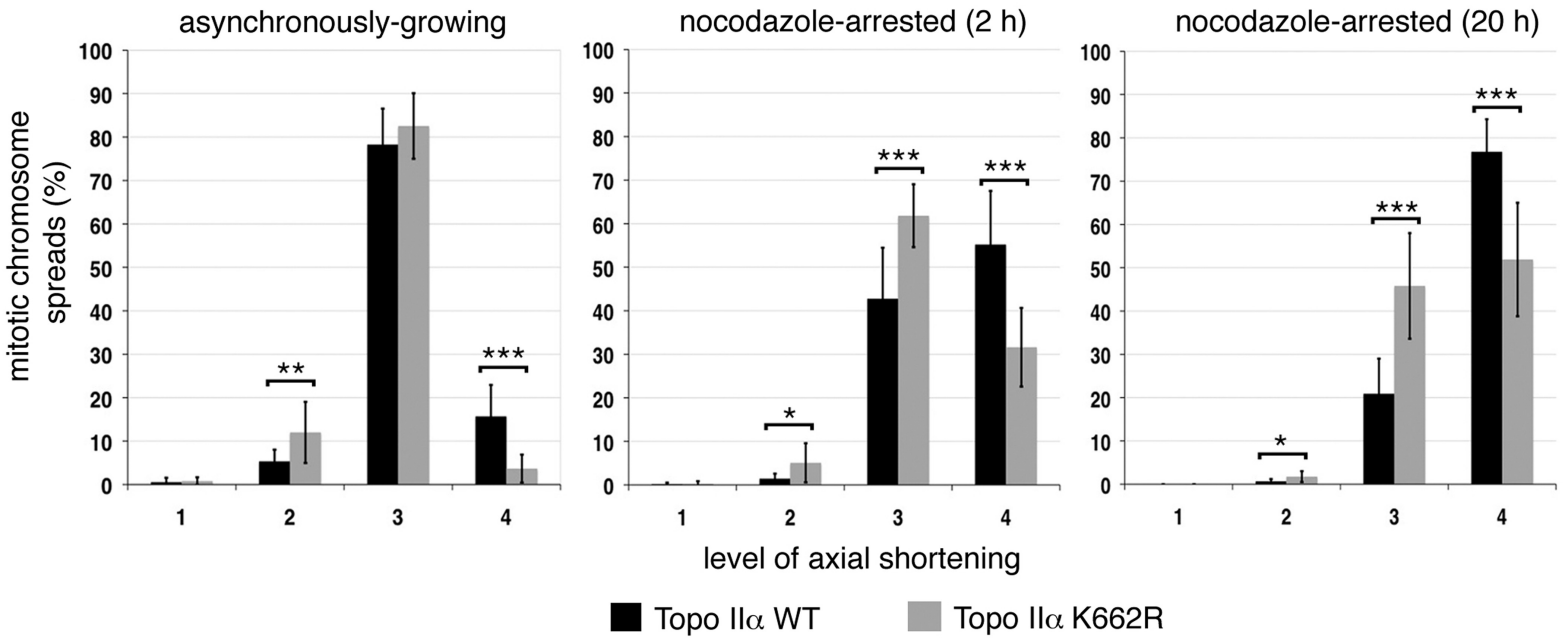
The effect of catalytically compromised topo IIα (K662R) on mitotic chromosome formation. Chromosome compaction status in independently derived HTETOP stable cell clones (n6) rescued from dox lethality by expression of topo IIα K662R (N-terminally Flag-tagged) was compared with stable cell clones expressing Flag:topo IIα WT (n3). The results from asynchronously growing cell populations are shown, together with those from cells held in M-phase by exposure to nocodazole for either 2 or 20 h. Each stable cell line was analyzed in ≥3 independent experiments, with ∼100 cells scored per experiment. The data sets for all clones expressing either K662R or WT topo IIα were combined. Data points represent the mean ±sd. The frequency of spreads showing the compact mass (CM) phenotype were <2% in all cases.

Mitotic chromosome formation was then examined in chromosome spreads from the HTETOP clones expressing topo IIα-K662R in place of WT (Figure 3). The frequency of spreads showing hypercompaction (level 4) was found to be consistently lower than in cells expressing WT protein. This differential was observed irrespective of whether the fixed mitotic cells were from asynchronously growing populations or cell populations that had been delayed in M-phase by exposure to nocodazole (for 2 or 20 h). Depletion of topo IIβ, using siRNA, had no additional impact on axial shortening in the topo IIα-K662R-expressing lines (data not shown). This indicates that topo IIα-K662R is significantly less efficient in mitotic chromosome formation.

### Hyperefficient chromosome shortening in M-phase following RO3306 arrest

**Figure 4.**
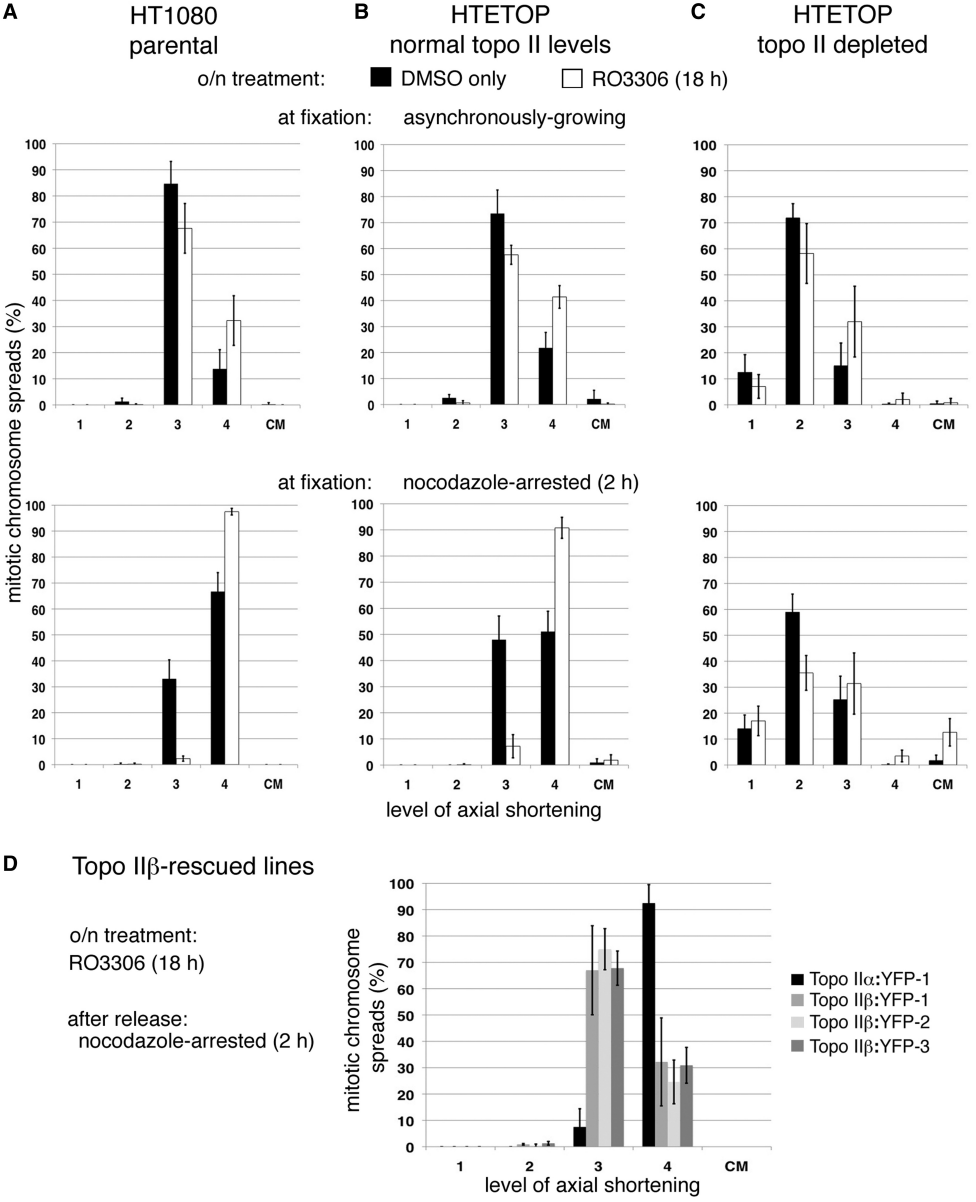
Enhanced hypercompaction following RO3306 arrest. Cells were delayed at the G2/M boundary by an overnight exposure to RO3306 (or treated with DMSO only). Following release back into the cell cycle, cells were treated with hypotonic and fixed in methanol: acetic acid, either when growing asynchronously (30 min post RO3306 washout) or after a nocodazole-induced delay (2 h) and mitotic chromosome formation assessed in (**A**) HT1080 cells (the parental cell line of HTETOP), (**B**) untreated HTETOP (normal levels of both topo II isoforms), (**C**) HTETOP cells depleted of both topo II isoforms (dox + siTopo IIβ, 72 h) and (**D**) HTETOP cells that have been rescued from dox lethality by expression of topo IIβ:YFP compared with those expressing topo IIα:YFP. All data points represent the mean (±sd) based on ≥3 independent experiments, with ∼100 cells scored per experiment.

Prolonged delay at the G2/M boundary, through the presence of the Cdk1 inhibitor RO3306 (50), leads to enhanced numbers of highly shortened stubby (level 4) chromosomes when either HTETOP or HT1080 cells are released into M-phase. This is especially pronounced in cells delayed in the ensuing M-phase (nocodazole 2 h), with chromosomes in 91–98% of spreads being hypercompacted, compared with 51–67% when solvent only treated cells are exposed to nocodazole (Figure 4A, B). This enhancement is dependent on topo II activity, as HTETOP cells depleted of both isoforms (dox + siTopoIIβ, 72 h) are unable to hypercompact their chromosomes when held in M-phase after RO3306 inhibition (Figure 4C). Under these conditions compact CMs were observed (∼12% of spreads). In cells rescued from dox lethality by overexpression of topo IIβ RO3306 does not induce any significant increase in the number of spreads with hypercompacted chromosomes, suggesting that this phenotype is dependent on the α isoform (Figure 4D compared with Figure 2C, *P =* 0.20).

### Generation of a human cell line from which topo IIα can be rapidly and reversibly depleted

**Figure 5.**
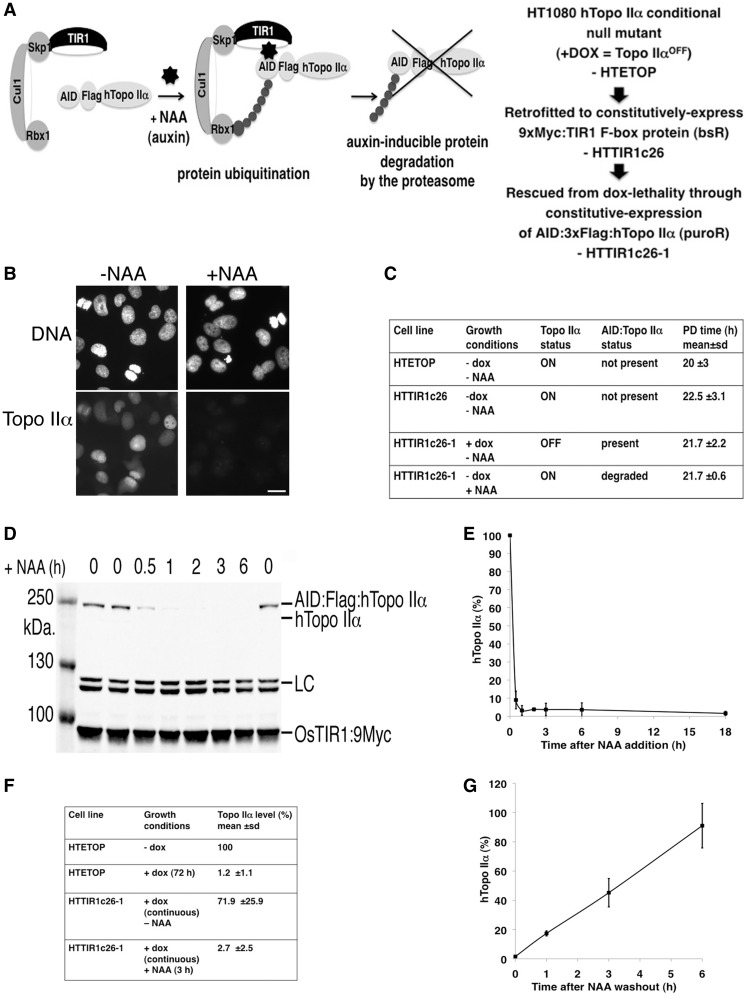
Rapid depletion of topo IIα from HTETOP cells. (**A**) Strategy used to generate an HTETOP derivative from which topo IIα can be rapidly depleted. (**B**) Indirect IF of topo IIα in HTTIR1c26-1 cells grown continuously in the presence of dox (to deplete untagged topo IIα) and blasticidin (to maintain TIR1 expression), with or without 1 mM NAA for 3 h (to degrade AID-tagged topo IIα). Topo IIα was detected using anti-topo IIα antibody (FITC). DNA was counterstained using DAPI. Scale bar, 25 µm. (**C**) Comparison of the PD times of the parental HTETOP cell line (-dox) with HTTIR1c26 (a derivative retrofitted to constitutively express TIR1) and HTTIR1c26-1 (which expresses both TIR1 and AID degron:Flag:Topo IIα). The growth of the latter clone was examined under both +dox/−NAA conditions (where the AID:Flag:Topo IIα fusion protein is the main form of topo IIα present in the cells) and under +NAA/−dox selection (when the bulk of the topo IIα present is untagged). (**D**) Fluorescence immunoblotting of topo IIα, myc-tagged TIR1 and an unidentified protein detected by the anti-topo IIα antibody (LC). (**E**) Graph showing topo IIα levels at various time points after addition of 1 mM NAA. Shown are mean values. Error bars represent sd. Each data point is based on <3 independent experiments. (**F**) Topo IIα level in the parental HTETOP cell line (− and + dox) compared with that in HTTIR1c26-1 after exposure to 1 mM NAA (3 h). (**G**) Graph showing the recovery of topo IIα levels at various time points after removal of 1 mM NAA. Shown are mean values. Error bars represent sd. Each data point is based on three independent experiments.

The dox-regulatable conditional null mutant used in this work is a powerful reagent. However, transcriptional regulation results in a slow decline of the protein-of-interest, a disadvantage when interpreting phenotypes arising from depletion of topo IIα, a protein required at multiple cell cycle stages. To study specifically the mitotic chromosome functions of topo IIα, new tools are required (24). Using the auxin-degron system (46), a derivative of HTETOP has been generated from which topo IIα can be rapidly and reversibly depleted (Figure 5). A clone constitutively expressing an myc-tagged F-box protein TIR1 (designated HTTIR1c26) was established and subsequently rescued from dox lethality by the introduction of constitutively expressed topo IIα tagged at its N-terminus by the AID degron and Flag epitope (designated HTTIR1c26-1) (Figure 5A). Indirect IF revealed population-wide disappearance of topo IIα (from ∼99% of cells) following exposure to both dox (continuous) and auxin (NAA, 3 h) (Figure 5B). The PD time of the various derivatives was similar (Figure 5C) suggesting that (i) the presence of the TIR1 protein is not detrimental to HTETOP cells; and (ii) untagged topo IIα (present in the absence of dox) and the AID:Flag:Topo IIα fusion protein (present in the absence of the synthetic auxin NAA) are equally competent at maintaining viability of the proliferating cells. Fluorescence immunoblotting showed that the AID-tagged topo IIα protein is maximally depleted from asynchronously growing cells by 1 h post-NAA addition to the culture medium (Figure 5D and E). A similar depletion profile was seen following addition of IAA (instead of NAA) (data not shown). The level of topo IIα protein in the various clones grown in the presence or absence of dox and/or NAA was compared with that of the parental HTETOP cell line (-dox) (Figure 5F). The extent of depletion after NAA treatment is slightly less than that achieved through doxycycline-induced repression of topo IIα transcription (protein depletion of 2.7% compared with 1.2%), but the speed of depletion is much more rapid (∼1 h compared with 48–72 h). Effective depletion was also detected in cells delayed at the G2/M border after overnight (∼18 h) exposure to RO3306 (depletion level: 1.5% ± 1.6). Moreover, NAA-induced topo IIα depletion is reversible (Figure 5G), although recovery of topo IIα protein levels is slower than depletion (reaching ∼45% that of untreated cells 3 h post-NAA removal and ∼91% by 6 h). Recovery of topo IIα protein levels was much poorer following exposure to IAA (data not shown).

### Rapid depletion of topo IIα confirms its late G2/M phase role in axial shortening

**Figure 6.**
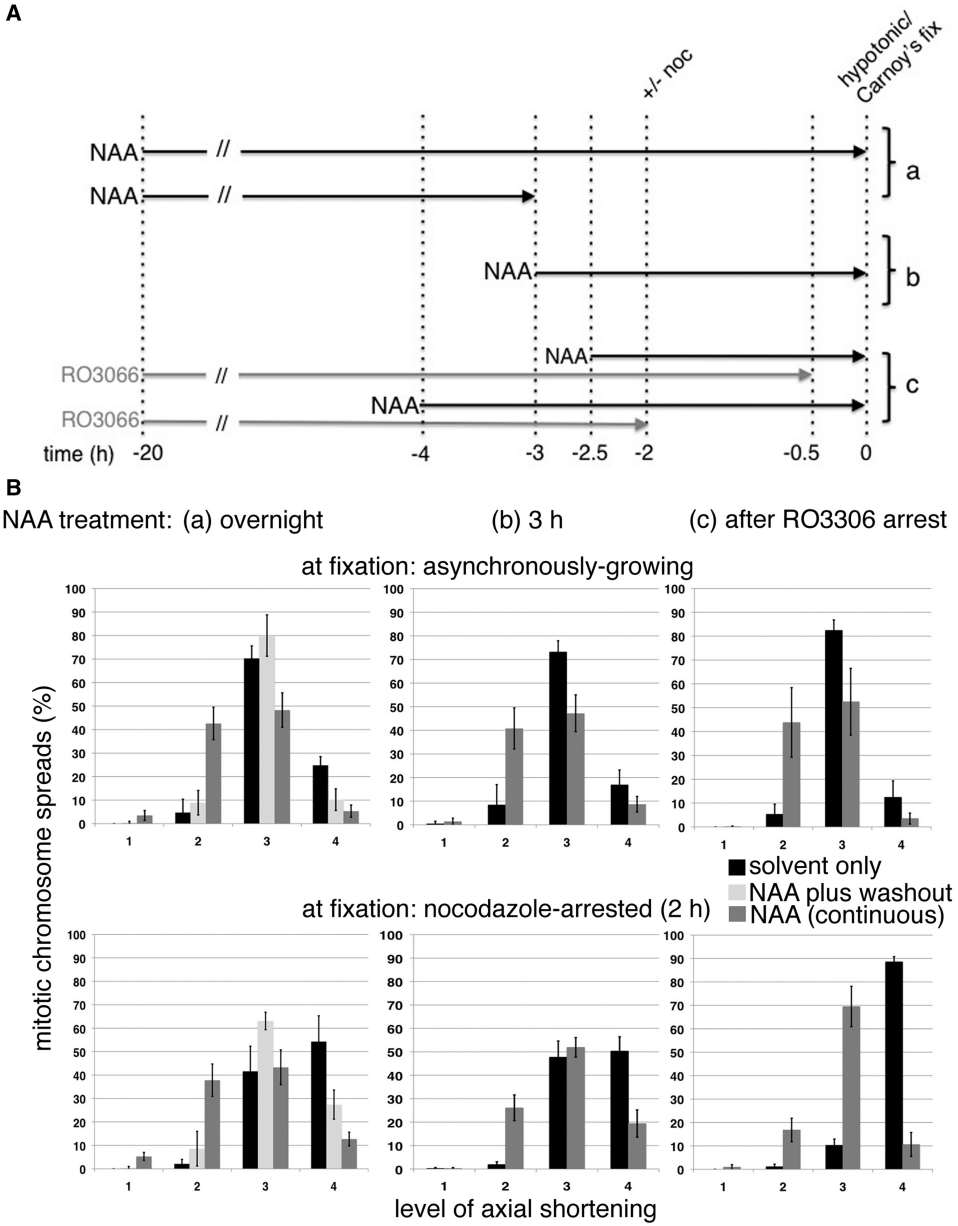
(**A**) Schematic outlining the experimental strategy used to compare the impact on mitotic chromosome formation of depleting topo IIα from late G2 only versus its continuous depletion throughout the cell cycle. In all experiments, cells were treated with siRNA (72 h) to deplete topo IIβ. (a), (b) and (c) refer to sections in 6B. (**B**) Assessment of mitotic chromosome formation following various NAA (or DMSO only) treatment regimens: (a) overnight, with or without a 3 h recovery period post NAA washout, (b) for 3 h and (c) cells delayed at the G2/M border by overnight treatment with RO3306 were exposed to NAA for 2 h (RO3306 + NAA) before release back into the cell cycle in the continued presence of NAA. Cells were treated with hypotonic and fixed in methanol–acetic acid either when growing asynchronously (cells arrested by RO3306 were fixed 30 min post RO3306 washout) or after M-phase arrest (nocodazole 2 h). For all conditions, data points represent the mean (±sd) based on ≥3 independent experiments, with ∼100 cells scored per experiment. The frequency of spreads showing the compact mass (CM) phenotype was <2.4% in all cases.

Mitotic chromosome formation was examined following NAA-induced topo IIα degradation in HTTIR1c26-1 cells pretreated with siTopo IIβ (Figure 6A). Prolonged depletion of topo IIα, by exposure to NAA overnight (∼20 h), resulted in a significant increase in longer thinner chromosomes and a decrease in hypercompacted spreads (Figure 6B). The phenotype is not as severe as that seen in HTETOP cells treated for 72 h with dox and siTopo IIβ (Figure 1C). If NAA is removed the following day (after ∼17 h exposure) and the cells allowed to recover for 3 h before fixation (with topo II protein levels returning to ∼50% normal; Figure 5G), the phenotype is extensively rescued: the frequency of spreads showing only level 2 compaction falls from 40% to <10%, while the number of cells with hypercompacted chromosomes increases [although it fails to reach that seen in cells with unperturbed topo IIα levels (Figure 6B)]. A virtually identical distribution to that induced by overnight NAA treatment is seen both in cells treated with NAA for 3 h only before fixation, and where cells arrested at the G2/M border (by prolonged inhibition of Cdk1 by RO3306) are exposed to NAA for 2 h before release back into the cell cycle (in the continued presence of NAA) (Figure 6B). These experiments suggest that depletion of topo IIα from late G2 has a significant impact on the cell’s ability to assemble mitotic chromosomes, resulting in an increase in the frequency of mitotic spreads with longer thinner chromosomes and a concomitant decrease in those showing hypercompaction. The enhancement in nocodazole-treated cells achieving level 4 compaction post-RO3306 arrest is once again absent from cells depleted of topo II (Figure 6B).

## DISCUSSION

Topo IIα is the major isoform of this enzyme present in cycling vertebrate cells. Many reports have shown that depletion of topo IIα impacts mitotic chromosome formation (41,42,44), and in human cells, we have shown previously that depletion of topo IIα results in an increased incidence of long, thin ribbon-like chromosomes, with a reduced interkinetochore distance under tension in metaphase (7,45). Moreover, these topo IIα-depleted chromosomes retain condensin and, based on an *in vitro* assay, their structural integrity seems unperturbed. This phenotype is virtually the mirror image of that seen when either condensin or KIF4A is depleted from chicken DT40 chromosomes (44,51–53). In these cases, chromosomes were reported to be fatter and wider than normal, to have an increased interkinetochore distance and to have a compromised inner structure. Recently, a model has been proposed in which condensin and KIF4 work in parallel to compact chromosomes laterally, whereas topo IIα acts in an opposing pathway to shorten chromosomes arms (44). In addition to enabling compaction through the removal of entanglements, topo II may also contribute to compaction by introducing entanglements (54). Meanwhile, its continued presence, as a major component of the compacted M-phase chromosome, both along the arms and at the centromere, may confer flexibility on the structure, as well as ensuring efficient removal of residual linkages and chromosome segregation.

In this study, we show that topo IIβ also contributes to mitotic chromosome formation in cycling human cells. Although normal levels of topo IIβ are unable to maintain the viability of proliferating cells in the absence of topo IIα, if the small amount of topo IIβ present in HTETOP cells (estimated to be ∼3% that of topo IIα) is simultaneously depleted alongside topo IIα, the impact on mitotic chromosome assembly is more severe than following depletion of topo IIα alone. This suggests that even a low level of topo II activity is sufficient for the initial stages of axial shortening during mitotic chromosome formation. However, a significant effect of topo IIα depletion, seen irrespective of the presence or absence of topo IIβ, is the failure of chromosomes to hypercompact when cells are delayed in M-phase. This failure of topo IIα-depleted chromosomes to hypercompact has been observed previously in chicken DT40 cells conditionally induced to express shTopo IIα (44) and suggests that hypercompaction in M-phase requires much higher levels of topo II activity.

Topo IIα has evolved to fulfill this function, both through its upregulation (4,5,55–57) in proliferating cells and through the presence of the α-CTR that enables this isoform to target mitotic chromatin efficiently (11). Topo IIβ can only promote hypercompaction of mitotic chromosomes when it is highly overexpressed (∼50- to 100-fold higher than normal). When overexpressed to this extent, topo IIβ protein levels are not dissimilar to topo IIα levels. However, topo IIβ promotes axial shortening much less efficiently than (similar amounts of) topo IIα (Figures 2C and 4D). Thus, assembly of the stubby hypercompacted chromosomes characteristic of cells delayed in M-phase requires high levels of topo II activity and is perturbed by effects that interfere with the enzyme’s action: such effects include reduction in topo IIα protein levels, perturbation of topo IIα activity through mutation of the catalytic domain (e.g. K662R) or substitution of α by high levels of the β isoform.

It was observed that prolonged treatment of cells with the Cdk inhibitor RO3306 results in extremely efficient axial shortening when cells are released back into the cell cycle. In the presence of normal topo IIα protein levels exposure to RO3306 results in ≥90% of spreads from cells delayed in M-phase for 2 h by nocodazole having short stubby chromosomes. This effect is dependent on topo II activity, as in cells depleted of both isoforms few cells achieve hypercompaction even following RO3306 exposure. Moreover, the RO3306 effect is dependent specifically on the α isoform, as no enhancement is seen in HTETOP cells rescued from dox-induced lethality by overexpression of topo IIβ. Fluorescent immunoblotting of asynchronously growing HTETOP and HT1080 cell populations compared with cells arrested by overnight exposure to RO3306 indicates similar topo IIα protein levels (data not shown). These observations could be explained either by progressive decatenation, throughout G2, by a constant level of topo II activity, with a delay in late G2 leading to more decatenation and hence axial shortening or, alternatively, by the activity of topo IIα protein in the cell increasing as the cell transits G2, with a prolonged delay in late G2 enhancing this effect. Various protein modifications and protein interactions have been suggested to influence topo II decatenation activity *in vitro*, although often little is known about their *in vivo* significance (49,57–69). Phosphorylation of topo IIα increases during M-phase and the sites phosphorylated change during the cell cycle (70–75). However, whether mitosis-specific phosphorylation of topo IIα influences chromosome formation remains unclear.

This report and that of Sameijima *et al.* (44) show that in vertebrate cells topo II activity plays an essential role in ongoing axial shortening during prometaphase and metaphase. In addition, in this study, the extent to which the long, thin, ribbon-like phenotype of topo IIα-depleted chromosomes arises from insufficient topo IIα activity during M-phase itself, as opposed to being a consequence of insufficient activity earlier in the cell cycle, has been addressed using auxin-inducible degradation. The decatenating action of topo II is important during many aspects of DNA metabolism in G1, S and G2 phases of the cell cycle. In particular, topo IIα is thought to play a crucial role in the termination of DNA replication (76–78). By the rapid depletion of topo IIα from late G2 onwards, we have been able to rule out effects caused by loss of topo IIα function earlier in the cell cycle and show that topo II activity during late G2 and M-phase is truly required for shaping mitotic chromosomes. The availability of a human cell line that allows for the rapid and reversible depletion of topo IIα will be a powerful tool for future studies into cell cycle stage-specific phenotypes.

## FUNDING

Medical Research Council [MRC DTG 2011] and Cancer Research-UK [C9609/A10506]. Funding for open access charge: University of Cambridge RCUK OA Block Fund.

*Conflict of interest statement*. None declared.
